# Electrocardiographic study in Ghanaian children with uncomplicated malaria, treated with artesunate-amodiaquine or artemether-lumefantrine

**DOI:** 10.1186/1475-2875-11-420

**Published:** 2012-12-17

**Authors:** George O Adjei, Collins Oduro-Boatey, Onike P Rodrigues, Lotte C Hoegberg, Michael Alifrangis, Jorgen A Kurtzhals, Bamenla Q Goka

**Affiliations:** 1 Centre for Tropical Clinical Pharmacology & Therapeutics, University of Ghana Medical School, College of Health Sciences, University of Ghana, Accra, Ghana; 2 Department of Child Health, University of Ghana Medical School, College of Health Sciences, University of Ghana, Accra, Ghana; 3 Centre for Medical Parasitology at Department of International Health, University of Copenhagen and Department of Infectious Diseases, Copenhagen University Hospital, Copenhagen, Denmark

**Keywords:** Malaria, Combination therapy, Cardiotoxicity, Children, Ghana

## Abstract

**Background:**

Several anti-malarial drugs are associated with adverse cardiovascular effects. These effects may be exacerbated when different anti-malarials are used in combination. There has been no report yet on the potential cardiac effects of the combination artesunate-amodiaquine.

**Methods:**

Electrocardiographic (ECG) intervals in Ghanaian children with uncomplicated malaria treated with artesunate-amodiaquine (n=47), were compared with that of children treated with artemether-lumefantrine (n=30). The ECG measurements were repeated one, two, three, seven and 28 days after treatment. The ECG intervals of artesunate-amodiaquine treated subjects were correlated with plasma concentrations of desethylamodiaquine (DEAQ), the main metabolite of amodiaquine.

**Results:**

The mean ECG intervals were similar in both groups before treatment. After treatment (day 3), ECG intervals changed significantly from baseline in all subjects, but there were no differences between the two treatment groups. A significantly higher proportion of children treated with artesunate-amodiaquine developed sinus bradycardia compared with artemether-lumefantrine treated subjects (7/47 *vs* 0/30; χ^2^ p=0.03). Subjects who developed bradycardia were significantly older, and had higher DEAQ concentrations than those who did not develop bradycardia. The proportion of subjects with QTc interval prolongations did not differ significantly between the groups, and no relationship between prolonged QTc intervals and DEAQ levels were observed. No clinically significant rhythm disturbances were observed in any of the subjects.

**Conclusion:**

Artesunate-amodiaquine treatment resulted in a higher incidence of sinus bradycardia than artemether-lumefantrine treatment in children with uncomplicated malaria, but no clinically significant rhythm disturbances were induced by combining artesunate with amodiaquine. These findings, although reassuring, may imply that non-amodiaquine based artemisinin combination therapy may be preferable for malaria treatment in patients who are otherwise at risk of cardiac effects.

## Background

Artesunate-amodiaquine combination is an efficacious artemisinin combination therapy (ACT) regimen that has been widely adopted for first-line treatment of uncomplicated malaria in many endemic countries [1-3]. There is limited information, however, on aspects of the safety of this combination therapy regimen, particularly on its potential cardiovascular effects.

Amodiaquine belongs to the 4-aminoquinoline class of anti-malarials, and has previously been associated with minor cardiac effects, including bradycardia, in adults [4]. Amodiaquine is also structurally related to chloroquine, a 4-aminoquinoline that has significant cardiac effects, including lethal cardiovascular toxicity in overdose [5,6].

The artemisinin derivatives on the other hand, are among the safest anti-malarials known; however, earlier studies in animals, especially of the oil-soluble derivatives have linked these anti-malarials with cardiac effects, including abnormalities in depolarization [7], or suppression of cardiac conduction [8].

These reported cardiovascular effects of amodiaquine or artemisinin or its derivatives are considered clinically insignificant, but there is the possibility that these effects could be potentiated when these anti-malarials are used in combination: e g, overlapping therapy of mefloquine and halofantrine has been shown to result in a QT interval prolongation greater than that of either drug alone [9,10], and the QT interval prolongation of quinine is enhanced by prior administration of artemether-lumefantrine [11].

These considerations make it important that potential cardiovascular effects of newly introduced anti-malarials should to be evaluated in combination. However, there have been no reports yet, on the potential cardiac effects of the artesunate-amodiaquine combination, in spite of the fact that other ACT regimens that have been widely deployed for malaria control in endemic areas, including artemether-lumefantrine [12,13], dihydroartemisinin-piperaquine [14,15], and artesunate-mefloquine [16], have all been extensively evaluated for cardiotoxic potential.

This study reports the electrocardiographic (ECG) changes in Ghanaian children with uncomplicated malaria treated with artesunate-amodiaquine, or artemether-lumefantrine. The ECG changes in the artesunate-amodiaquine treated children have been correlated with plasma concentrations of desethylamodiaquine (DEAQ), the main, active metabolite of amodiaquine.

## Methods

### Study site and patients

The study was part of a clinical trial that was conducted to evaluate the safety and efficacy of artesunate-amodiaquine and artemether-lumefantrine as potential ACT regimens for first-line treatment of uncomplicated malaria in Ghana. Approval for the study was granted by the Ethics and Protocol Review Committee of the University of Ghana Medical School, and written, informed consent was obtained from the accompanying parent or guardian of all enrolled children. The full description of the study site and results of the trial have been previously reported [1]. Briefly, enrolled children aged 0.5–14 years with uncomplicated malaria were treated with: i) artesunate (Plasmotrim®, Mepha; Switzerland), 4 mg/kg body weight as a single daily dose + amodiaquine (Camoquine®, Pfizer; Dakar, Senegal), 10 mg/kg body weight, single daily dose, for three days; or, ii) artemether-lumefantrine (Coartem®, Novartis Pharma AG, Basel, Switzerland; 20 mg artemether and 120 mg lumefantrine), given at zero and eight hours on the first day and then twice daily for the two subsequent days according to body weight: 9–14 kg, one tablet/dose; 15–24 kg, two tablets/dose; 25–34 kg, three tablets/dose; 35 kg and over, four tablets/dose.

After enrolment, a full clinical examination was done, and key demographic and clinical information were recorded on a standard questionnaire. Venous blood was collected into EDTA and heparinized tubes for the determination of parasite count, haematological and biochemical investigations, and for amodiaquine and plasma drug (amodiaquine and metabolites; desethylamodiaquine, bis-desethylamodiaquine) concentration measurements.

### Electrocardiography (ECG)

A standard 12-lead ECG (Esaote P80, Firenze, Italy) was performed at a paper speed of 50 mm/s and a sensitivity of 10 mm/mV. The ECG was done at baseline (0 h), 24, and 48 hours later, and then on days 3, 7 and 28 in children who were able to cooperate with the testing. The 24 and 48 hour times were chosen to minimize the possible effect of circadian variation on ECG indices [17], and the day 3 ECG approximates the time of expected high plasma concentrations of DEAQ. The ECGs were evaluated with respect to rate, rhythm, QT interval, QRS interval, as well as qualitative changes in T and U wave morphology. The PR interval was measured from the onset of the P wave to that of the R wave, the QT intervals were measured from the onset of the QRS to the end of the T wave (defined as return of the terminal limb to baseline), and the RR interval was measured as the time between the peaks of two QRS complexes. The QT interval was measured in lead II or in lead III or V5 in case of artefacts (low T wave amplitude) in lead II. The corrected QT interval (QTc) was calculated, using i) Hodges’ formula: (QTc_H_) = QT + 1.75 (ventricular rate - 60); ii) Bazett’s formula: (QTc_B_) = QT/RR^0.5^); and iii) Fridericia’s formula: (QTc_F_) = QT/RR^0.3^).

### Laboratory investigations

Haemoglobin concentration and total white blood cell count (WBC) were determined by means of an automated analyzer (Cell Dyn, Abbott Laboratories, USA). Thick and thin blood films were stained with Giemsa and read under 100X magnification. Parasite density was determined by counting the number of asexual parasites per 200 WBCs and multiplied by the measured WBC count to obtain a count per microlitre.

### Plasma amodiaquine and metabolite concentrations

Plasma concentrations of amodiaquine and its main metabolites were measured from samples taken before (on day 0) or after (on days 3, 7, and 28) ECG measurements, using a reverse-phase high performance liquid chromatographic method with ultraviolet detection. The recovery for the drugs in plasma was 81%, 94% and 96% for bis-desethylamodiaquine, desethylamodiaquine, and amodiaquine, respectively.

### Data analysis

The data were analysed using Stata™ (version 10, Stata Corp, Texas, USA). Continuous data were analysed, using the paired or unpaired T tests for normally distributed data, and the Mann–Whitney U tests for non-normally distributed data, as appropriate. Categorical data were analysed using the Chi square or Fischer exact test, with Yates correction as appropriate. Categorical analyses of outlying QTc values were done to ascertain the proportion of patients with: i) absolute QTc intervals of >30 ms, or 60 ms from baseline, and ii) QTc increases >25% from baseline, and absolute QTc intervals >440 ms. The relationship(s) between specified variables was analysed using Spearman’s correlation or by linear regression. P values <0.05 were considered significant.

## Results

### Comparisons of mean ECG interval changes

The baseline demographic and selected clinical parameters on admission (day 0) for the two treatment groups were similar (Table 1). There was a difference in the mean ECG parameters before (day 0) and immediately after (day 3) treatment, within each of the treatment groups (p<0.01). However, there were no significant differences between the ECG intervals in the two groups on days 3, or day 7 (Table 2). The proportion of subjects with bradycardia (defined as a ventricular rate below the normal for age), was significantly higher (p=0.03) in the artesunate-amodiaquine group (14.8%; 7/47), compared with the artemether-lumefantrine group (0/30). The mean age of subjects who developed bradycardia (11.8 years) was significantly higher (p<0.001) than those who did not (6.3 years). Selected characteristics of subjects who developed bradycardia are shown (Table 3). The proportion of subjects with QTc interval change greater than 30 ms from baseline was also higher in the artesunate-amodiaquine group (31.9%; 15/47) compared with the artemether-lumefantrine group (20%; 6/30), but the difference was not statistically significant (OR, 1.9, 95% CI, 0.6-6.4, p=0.25), and this was not confined to older age groups. Selected characteristics of subjects with QTc increase more than 60 ms from baseline are shown (Table 4).

**Table 1:** Baseline characteristics

	**Artemether-lumefantrine (n=30)**	**Artesunate-Amodiaquine (n=47)**
*Age (yrs)	8.00 (4–12)	7.00 (1.5-14)
Weight (Kg)	24.00 (8.70)	23.28 (13.69)
Parasite density (/μL)	76545 (68726)	84746 (108063)
Haemoglobin (g/dL)	12.22 (1.59)	11.51 (1.94)
WBC (x10^3^/L)	8.42 (3.43)	9.02 (3.72)
Rate (min)	114.73 (19.26)	119.53 (21.15)
PR (ms)	124.00 (16.10)	126.81 (15.20)
QT (ms)	296.00 (24.86)	286.81 (38.51)
QTc_H_ (ms)	391.80 (24.40)	390.28 (26.59)
QTc_B_ (ms)	406.03 (27.84)	399.57 (33.44)
QTc_F_ (ms)	365.16 (23.11)	357.42 (33.27)

Data are means and standard deviations except *age (range); P > 0.05 all comparisons.

**Table 2:** ECG parameters [means, (SD)] on days 3 (upper panels) and day 7 (lower panels) for the two groups

	**Artemether-lumefantrine (n=30)**	**Artesunate-amodiaquine (n=47)**
Rate (min)	86.10 (14.89)	80.06 (15.76)
	90.11 (15.99)	90.11 (22.00)
PR (ms)	126.90 (18.73)	134.47 (21.14)
	134.29 (19.52)	130.91 (18.02)
QT (ms)	330.67 (29.59)	339.57 (29.92)
	321.43 (33.52)	323.56 (30.54)
QTc_H_ (ms)	376.47 (22.66)	374.81 (21.77)
	374.18 (19.19)	372.09 (21.94)
QTc_B_ (ms)	392.71 (27.99)	388.04 (26.70)
	389.36 (23.56)	387.67 (27.20)
QTc_F_ (ms)	370.54 (24.72)	370.77 (22.20)
	364.90 (22.85)	364.71 (25.040

QTc_B_ =Bazett’s corrected QTc; QTc_F_ =Fridericia’s corrected QTc; QTc_H_ = Hodges’ corrected QTc.

**Table 3:** Selected characteristics of subjects who developed bradycardia (HR < normal for age)

**Age (yrs)**	**Sex**	**AR day 0 (min)**	**AR day 3 (min)**	**Drug**	**DEAQ (ng/ml)**
9	Female	105	60	A-A	NA
12	Male	97	62	A-A	180
12	Male	81	58	A-A	346
12	Male	112	57	A-A	375
12	Female	109	57	A-A	NA
12	Male	100	56	A-A	NA
14	Female	88	56	A-A	324

AR=apex rate; DEAQ=desethylamodiaquine; NA=Not available; A-A=artesunate-amodiaquine; DEAQ=desethylamodiaquine concentration.

**Table 4:** Subjects with QTc >60 ms from baseline based on different QT correction formulae

**Age (yrs)**	**Sex**	**Drug**	**QTc day 0 (ms)**	**QTc day 3 (ms)**	Δ **QTc**_**(day3-day0)**_	**DEAQ (ng/ml)**
3.5	Male	A-A	361B	413B	52	109
			315F	379F	64	
			373H	390H	17	
5.5	Female	A-A	353B	399B	46	162
			310F	386F	76	
			363H	385H	22	
6.0	Female	A-A	404B	464B	60	0
			357F	426F	69	
			394H	430H	36	
6.5	Male	A-A	361B	443B	82	241
			315F	397F	82	
			373H	416H	43	
9.0	Male	A-L	366B	443B	77	NA
			335F	413F	78	
			355H	414H	59	
10.0	Female	A-L	387B	450B	63	NA
			347F	418F	71	
			376H	419H	43	

B=Bazett’s QTc; F=Fridericia’s QTC; H=Hodges QTc; ΔQTc =change in QTc; A-A=artesunate-amodiaquine; A-L=artemether-lumefantrine; DEAQ=desethylamodiaquine concentration; NA=Not applicable.

### DEAQ plasma concentrations and ECG changes (amodiaquine-based treatment groups)

Plasma DEAQ concentrations were available for 36 subjects in the artesunate-amodiaquine group. The mean DEAQ concentrations were, 194.51 (range, 0–375 ng/ml). The mean plasma DEAQ concentration of subjects who developed bradycardia (271 ng/ml) was significantly higher (p=0.01), than the mean DEAQ concentration of those who did not (165.2 ng/ml).

The correlation between DEAQ concentrations and day 3 QTc intervals, or between DEAQ concentrations and QTc change (between day 0 and day 3) was only weakly negative (data not shown). There was no marked trend between a plot of mean QTc on day 3 and plasma DEAQ concentrations (figure not shown).

### Comparisons based on the various QT correction formulae

The number of subjects with QTc interval prolongation >30 ms from baseline identified by the various correction formulae were: Bazett’s, (n=23); Fridericia’s, (n=21); and Hodges, (n=10). The corresponding number of subjects with QTc interval prolongation >60 ms from baseline identified were: Bazett’s, (n=4); Fridericia’s, (n=6), and Hodges, (n=0). The correlation between the QTc intervals, corrected using any of the three formulae, and RR intervals were only weakly negative (Table 5). A univariate linear regression model identified day 0 QTc intervals as the only significant predictor of the day 3 QTc interval.

**Table 5:** Correlation between QTc and RR, using the various correction formulae

**Hodges formula**	**QTC**	**RR**	**Pearson’s correlation coefficient**
QTC vs RR day 0 (A-L)	391.80 (24.40)	0.54 (0.09)	−0.675
QTC vs RR day 3 (A-L)	376.47 (22.66)	0.72 (0.12)	−0.256
QTC vs RR day 7 (A-L)	374.18 (19.19)	0.69 (0.14)	0.065
QTC vs RR day 0 (A-A)	390.28 (26.59)	0.52 (0.10)	−0.252
QTC vs RR day 3 (A-A)	374.81 (21.77)	0.78 (0.15)	−0.239
QTC vs RR day 7 (A-A)	372.09 (21.94)	0.70 (0.12)	0.458
**Bazett’s formula**	**QTc**	**RR**	**Correlation coefficient**
QTCb vs RR day 0 (A-L)	406.03 (27.84)	0.54 (0.09)	−0.373
QTCb vs RR day 3 (A-L)	392.71 (27.99)	0.72 (0.12)	−0.366
QTCb vs RR day 7 (A-L)	389.36 (23.56)	0.69 (0.14)	−0.14
QTCb vs RR day 0 (A-A)	399.57 (33.44)	0.52 (0.10)	0.097
QTCb vs RR day 3 (A-A)	388.04 (26.70)	0.78 (0.15)	−0.492
QTCb vs RR day 7 (A-A)	387.67 (27.20)	0.70 (0.12)	−0.283
**Fridericia’s formula**	**QTc**	**RR**	**Correlation coefficient**
QTCf vs RR day 0 (A-L)	365.16 (23.11)	0.54 (0.09)	0.026
QTCf vs RR day 3 (A-L)	370.54 (24.72)	0.72 (0.12)	0.032
QTCf vs RR day 7 (A-L)	364.90 (22.85)	0.69 (0.14)	0.364
QTCf vs RR day 0 (A-A)	357.42 (33.27)	0.52 (0.10)	0.419
QTCf vs RR day 3 (A-A)	370.77 (22.20)	0.78 (0.15)	−0.023
QTCf vs RR day 7 (A-A)	364.71 (25.04)	0.70 (0.12)	0.116

QTcb =Bazett’s corrected QTc; QTcf =Fridericia’s corrected QTc; QTch = Hodges’ corrected QTc; A-A=artesunate-amodiaquine; A-L=artemether-lumefantrine; RR=RR interval.

### Other ECG changes

The pre-treatment ECG of a 12 year-old girl (artesunate-amodiaquine-treated) showed features of right QRS axis deviation. This subject developed bradycardia on day 3 (ventricular rates were, 109, 57, 68, 71, on days 0, 3, 7 and 28, respectively but, there were no associated adverse cardiovascular effects.

## Discussion

The importance of evaluating potential cardiotoxic effects of newly introduced anti-malarial drugs has been highlighted with discovery of the cardiotoxicity of halofantrine after its registration and introduction into clinical practice. Apart from halofantrine, anti-malarials, such as quinine and quinidine, have also been associated with cardiotoxicity, mainly clinically significant delays in ventricular repolarization, which is reflected on the ECG as prolongation of the QT interval. The findings of relatively short pre-treatment QT intervals in this study is consistent with reports from ECG studies in African children with uncomplicated malaria [18], and are presumed to result from differences in autonomic state between acute illness and recovery. It is suggested that the increased sympathetic tone, blunted autonomic postural responses, and faster heart rates during acute malaria accelerates cardiac conduction, leading to a shortening of the QT interval. These changes, when reversed during convalescence then result in QT interval prolongation [reviewed in [19].

The observed difference in bradycardia occurrence between the amodiaquine-artesunate and artemether-lumefantrine groups is likely a reflection of a true difference between the treatment regimens. This is because the major differences between childhood and adult ECG (e.g., faster ventricular rate that slows with age, dimunition of right ventricular dominance, leftward shift of QRS axis with age) are related to maturational changes that may not necessarily impact directly on conduction. Furthermore, bradycardia occurred mostly in older children, similar to the previously reported high bradycardia incidence in amodiaquine-treated adults [4,20], and also consistent with findings from animal studies that showed that amodiaquine slows cardiac conduction [21,22]. Since alterations in cardiac conduction pathways are an important mechanism for pro-arrhythmic events, and bradycardia may by itself, precipitate long QT syndrome [23,24], the potential implications of artesunate-amodiaquine associated bradycardia merits further investigation.

The lack of association between plasma DEAQ levels and QTc interval prolongation in the amodiaquine-artesunate group could be due to lack of a consistent effect of amodiaquine on cardiac repolarization. This assertion is supported in part by the finding that baseline QTc intervals were the sole predictor of post-treatment QTc intervals. This lack of dose-related association may also be due to the fact that these effects of amodiaquine-based treatment differ from that of anti-malarials such as halofantrine [12,25] or quinine [26-28] whose cardiotoxic effects have been shown to be clearly dose-dependent.

The mean QTc interval changes from baseline were below the 25% increase beyond which such changes are considered of clinical concern [29], and the proportion of children with absolute QTc interval prolongation beyond 60 ms (a threshold that is considered significant for evaluating potential cardiotoxicity of new drugs), was higher in the artesunate-amodiaquine treatment group, though the number of subjects with these changes were low to allow meaningful statistical testing.

The Fridericia’s formula was as sensitive as the Bazett’s formula in identifying prolonged QT intervals in this study population. The utility of the Fridericia’s formula for evaluating potential cardiac effects of anti-malarial drugs in childhood studies could be further evaluated, since this correction formula is less rate-dependent, and other correction methods developed primarily for adults may not be applicable to childhood populations [30]. However, the weak correlation between the RR and QT intervals, corrected using the various formulae suggests that the different rate correction formulae had the desired effect.

Assessing the effect of antimalarials on an ECG, when used during the treatment of malaria could be problematic. This is because disease-associated changes in malaria confound QT interval measurements, and comparison of ECG changes should be done at the same heart rate, ideally in volunteers without intercurrent illness. The opportunities for such studies however, are limited. Furthermore, any potential disease-drug interactions would be impossible to detect in volunteer populations. The findings from this study are therefore, important not least because: i) there is paucity of published data on the ECG effects of anti-malarials, particularly in children, and ii) the artemether-lumefantrine group provides an exceptionally good reference standard, since this ACT regimen has been extensively evaluated and shown not to have any significant cardiotoxicity [12,13].

In conclusion, the standard ECG intervals of children with uncomplicated malaria treated with artesunate-amodiaquine or artemether-lumefantrine showed changes that are consistent with those observed in acute malaria. The higher incidence of sinus bradycardia in artesunate-amodiaquine treated subjects may have implications for concomitant use of this ACT with cardiac drugs, or for treatment of patients at increased risk of cardiac dysrhythmiae. The artesunate-amodiaquine regimen appears safe in other patients in this limited study, but further studies in a larger cohort are warranted for conclusive evidence on safety.

## Competing interests

The funding agencies had no role in the study design, collection, analysis, and interpretation of data, manuscript preparation or in the decision to submit for publication. The authors declare that they have no competing interests.

## Authors’ contributions

The study was designed by GOA, BQG, OPR and JK. The clinical work was done by GOA, BQG, OPR and CO-B. The laboratory work was done by GOA, LCH, MA, and JK. The data were analysed by GOA and JK. The manuscript was drafted by GOA, and all authors contributed significantly to the final draft. All authors read and approved the final manuscript.
